# Differentiation of Patients with Balance Insufficiency (Vestibular Hypofunction) versus Normal Subjects Using a Low-Cost Small Wireless Wearable Gait Sensor

**DOI:** 10.3390/bios9010029

**Published:** 2019-02-26

**Authors:** Tam Q. Nguyen, Jonathan H. Young, Amanda Rodriguez, Steven Zupancic, Donald Y.C. Lie

**Affiliations:** 1 Department of Otolaryngology, Texas Tech University Health Sciences Center, Lubbock, TX 79430, USA; tam.nguyen@ttuhsc.edu (T.Q.N.); steven.zupancic@ttuhsc.edu (S.Z.); 2 Department of Electrical & Computer Engineering, Texas Tech University, Lubbock, TX 79409, USA; 3 School of Medicine, Texas Tech University Health Sciences Center, Lubbock, TX 79430, USA; jonathan.h.young@ttuhsc.edu; 4 Department of Special Education and Communication Disorders, University of Nebraska-Lincoln, Lincoln, NE 68583, USA; amanda.rodriguez@unl.edu

**Keywords:** dynamic gait index (DGI) tests, fall-risk prediction, fall prevention, wireless gait analysis sensor (WGAS), machine learning

## Abstract

Balance disorders present a significant healthcare burden due to the potential for hospitalization or complications for the patient, especially among the elderly population when considering intangible losses such as quality of life, morbidities, and mortalities. This work is a continuation of our earlier works where we now examine feature extraction methodology on Dynamic Gait Index (DGI) tests and machine learning classifiers to differentiate patients with balance problems versus normal subjects on an expanded cohort of 60 patients. All data was obtained using our custom designed low-cost wireless gait analysis sensor (WGAS) containing a basic inertial measurement unit (IMU) worn by each subject during the DGI tests. The raw gait data is wirelessly transmitted from the WGAS for real-time gait data collection and analysis. Here we demonstrate predictive classifiers that achieve high accuracy, sensitivity, and specificity in distinguishing abnormal from normal gaits. These results show that gait data collected from our very low-cost wearable wireless gait sensor can effectively differentiate patients with balance disorders from normal subjects in real-time using various classifiers. Our ultimate goal is to be able to use a remote sensor such as the WGAS to accurately stratify an individual’s risk for falls.

## 1. Introduction

Patients presenting with balance insufficiency or vestibular hypofunction may be at risk for injuries from falling. Fall injuries resulted in 31 billion dollars of annual health care expenditures and income losses in the United States alone [1,2]. From a clinical standpoint, the correlation between vestibular hypofunction and fall risks is readily apparent. It is the standard of care that all patients with diagnoses of peripheral weakness should be advised of fall risks and referred for vestibular rehabilitation [3]. The mechanism underlying balance instability predisposing an individual to falling is multifactorial, with muscular, neurologic, or visual etiologies. The ability to differentiate patients with balance insufficiency or vestibular hypofunction versus individuals with normal gaits is important to identify patients at risk for falling. Consequently, the development of predictive classifiers to achieve such identification would be desirable in alerting clinicians to take preventive measures and prophylactic treatment to mitigate fall risks, as well as to address the etiology of balance problems in patients earlier.

The measurement of gait has a long history, extending decades back to the developing of image-based tracking of walking motions [4]. Historically, gait measurement was relatively cumbersome, involving a number of parameters including kinetic analysis of joint movements through automated tracking systems and measurements of energy consumption [5]. A central consideration in gait analysis is determining the variables or features to measure in a walking motion. The feasibility of determining gait cycle phases from an accelerometer was established in [6], and similarly the estimation of initial and terminal contact during walking from accelerometer measurements was demonstrated in [7]. Similarly, the defining events in walking—heel strike, toe strike, heel-off, and toe-off—were automatically extracted, again using accelerometers [8]. Such measurements of walking motions have been used to categorize gaits as normal or abnormal [9]. Aside from the determination of gait phases or events, other studies have turned to direct measurement of angular velocity and linear acceleration in categorizing normal and abnormal gaits [10].

In our efforts to evaluate predictive modeling of fall risk, machine learning is applied to classify patients as having either a normal or abnormal gait on the basis of measurements from a small low-cost wearable wireless gait analysis sensor (WGAS). The WGAS is a noninvasive custom built device worn by each individual subject. The study group was divided into a cohort of healthy volunteers and patients referred for vestibular hypofunction. It is our understanding that many classifier platforms have been pursued in the past with promising outcomes. However, attempts to correlate with standard battery vestibular and balance tests (for example videonystagmography, caloric tests, rotary chair, or dynamic platform posturography) used in clinical settings have not been uniformly pursued. The long-term aim is to determine the applicability of WGAS for fall prediction using classifiers that demonstrate high performance on the patient cohort, thus suggesting that accurate predictions would be made on patients presenting for evaluation of vestibular hypofunction.

## 2. Materials and Methods

### 2.1. Gait Data Collection

A low-cost and custom designed wireless gait analysis sensor (WGAS) collected gait data from patients with normal and abnormal gait movements as previously described [10,11,12,13]. Briefly, the WGAS consisting of a linear accelerometer integrated circuit (IC), a single-axis gyroscope, and a dual-axis gyroscope IC, and they are connected to a Texas Instruments (TI) MSP430 microprocessor to measure linear acceleration and rotation in three axes. The ICs are housed in a 3-D printed case manufactured with acrylonitrile butadiene styrene (ABS) plastic. Measurements from the WGAS were transmitted wirelessly with the TI SimpliciTI™ protocol through a 2.4 GHz USB transceiver to a receiver on a computer running LabVIEW™ (National Instruments). SimpliciTI is a TI proprietary low-power RF network protocol. It is low-cost, flexible, simple, and versatile to be used for MSP430+ and CC2500 2.4-GHz RF transceivers. The programmable data rate is up to 500 kbps with low current consumption. The WGAS sensor specifications are listed in Table 1.

**Figure 1:**
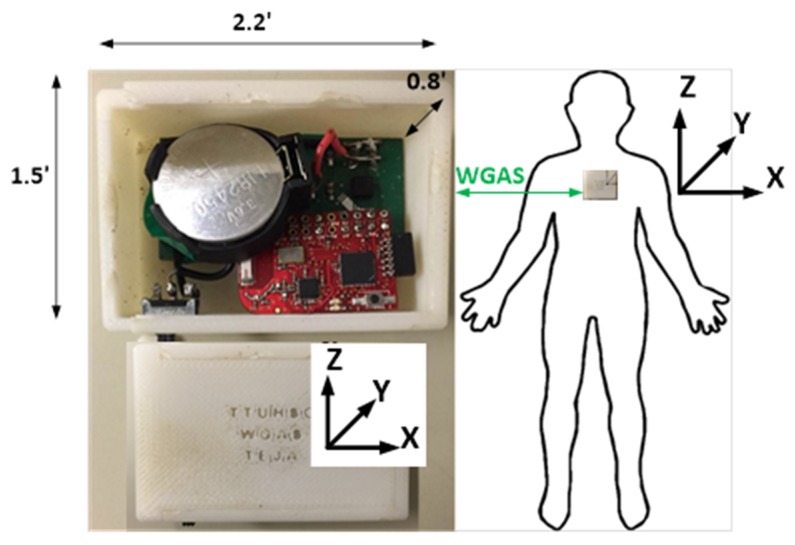
A diagram of the wireless gait analysis sensor is shown with dimensions and the axis orientation. The sensor is worn by subjects on the back at the T4 level.

Both accelerometer and gyroscope data were sampled at 160 Hz and digitized to 8 bits. The accelerometer (ST Microelectronics LIS344ALH) is a single 3 axis. The single axis gyroscope is a ST Microelectronics LY330ALH IC, and the dual-axis gyroscope is a ST Microelectronics LPR430AL IC. The output of the accelerometer was also scaled to ±6 g at ΔV = ±6 g/VDD (VDD, Supply voltage = 3.6 V) and multiplied by a sensitivity value of 3.752 mV/g for each axis. Similarly, the gyroscope output was scaled to 300°/s (dps) at ΔV = ±300 dps and multiplied by a sensitivity value of 3.33 mV/dps. Each patient wore a WGAS on the back at the T4 vertebral level while executing dynamic gait index (DGI) movements under various conditions. A diagram of the sensor is shown in Figure 1, and details of the DGI tests are listed in Table 2. All experimental protocols were reviewed and approved by the Texas Tech University Health Sciences Center Institutional Review Board (IRB).

The gait data was collected from patients with either normal or abnormal peripheral vestibular function, as measured by standard clinical assessments (i.e., caloric testing, vestibular evoked myogenic potentials, and/or rotational chair). The inclusion criteria for the patient cohort stipulated that the individuals were over 18 years of age and were either volunteers with no vestibular impairment or referred to the Texas Tech University Health Sciences Center (TTUHSC) with impaired vestibular function. Exclusion criteria disallowed individuals having current history of lower extremity orthopedic impairment, injury, or discomfort, or who were pregnant or terminally ill. The sample size was determined by the practical constraint of recruiting as many subjects as possible. The diagnosis of the patients with abnormal vestibular function was defined as unilateral or bilateral peripheral vestibular hypofunction that was considered in a chronic state (i.e., greater than six months since the onset of dizziness or imbalance symptoms). Patients in this group also perceived a reduction in their balance abilities throughout daily activities as indicated by the Dizziness Handicap Inventory, a questionnaire of functional performance for adults with dizziness and balance concerns [14].

### 2.2. Gait Data Analysis

Gait data was analyzed for each group and preprocessed into a single data frame. Each row in the data frame is a single sample that corresponds to a patient performing a particular DGI test, and each column corresponds to a single feature. There are six features in total: angular velocity in the *x*, *y* and *z* axes and linear acceleration along the *x*, *y*, and *z* axes. One additional column of the data frame also consists of assigned class labels, with the labels 0 and 1 assigned to normal and abnormal gaits, respectively. Each entry in the data frame is the range (difference between maximum and minimum) of the voltage values measured by the wireless gait sensor for the corresponding feature and sample (Equations (1) and (2), where $R_{A}$ is the range of linear acceleration and $R_{\omega }$ is the range of angular velocity):

(1) $$R_{A,\;x}=\max A_{x}-\min A_{x};\;R_{A,y}=\max A_{y}-\min A_{y};\;R_{A,z}=\max A_{z}-\min A_{z},$$

(2) $$R_{\omega ,\;x}=\max\omega_{x}-\min\omega_{x};\;R_{\omega ,\;y}=\max\omega_{y}-\min\omega_{y};\;R_{\omega ,\;z}=\max\omega_{z}-\min\omega_{z}.$$

Voltage values of zero were disregarded, as including such values would not capture the actual ranges of linear acceleration and angular velocity for subjects performing DGI tests. A zero voltage indicates no detectable movement for signal generation in a particular dimension and time. The resulting data frame then serves as the input to training and testing of the machine learning classifiers described below.

Principal components analysis (PCA) [15] was conducted for dimensionality reduction of the six-dimensional WGAS dataset. The dataset was centered to mean zero in each feature dimension but with no feature scaling. After PCA, the first three principal components (PC) were retained and used to visualize the dataset in PC space. Subsequently, the dataset was randomly divided into a training and test set, with the training set comprising approximately 80% of the full dataset. The random 80–20% train–test split of the WGAS dataset was then used to train machine learning classifiers to distinguish normal from abnormal gaits. An 80–20% train–test split allocates a large proportion of the dataset available for training; a test set with a small number of samples would lead to increased variance of the performance statistic.

A support vector machine (SVM) classifier [16] was trained by 3-fold cross-validation (CV) on the training set to select the optimal hyperparameters through grid search. All possible combinations of the choice of kernel (linear or radial basis function) and chosen values of the penalty parameter *C* of the error term were evaluated. To adjust for class imbalance, two penalty parameters for each of the classes (normal and abnormal) were weighted according to class frequencies as $\frac{Cn}{kn_{i}}$, where *n* is the total number of samples, *k* is the number of classes, and *n_i_* is the number of samples in class *i*. The distance of each test set sample to the separating hyperplane was calculated to construct a receiver operating characteristic (ROC) curve.

To employ dimensionality reduction as a preprocessing step for SVM training, PCA was applied to the training set with mean centering and no scaling. The resulting projection matrix consisted of loading vectors corresponding to the *n* largest eigenvalues of the feature covariance matrix, where *n* is the number of PCs retained. Multiplying the original centered data by the projection matrix yields the projection onto the *n*-dimensional subspace.

An artificial neural network (ANN) classifier was also trained by further dividing the training set into another 80–20% train–validation split, where the validation set was used to evaluate hyperparameters of number of layers and number of neurons in each layer of the ANN. The ANN is fully connected with the logistic sigmoid function as the activation function. The log-loss cost function was minimized through Adam optimization [17] with the learning rate fixed at 0.01. To adjust for class imbalance, the majority class (normal patients) was randomly undersampled from the training set. For both SVM and ANN, the training and testing sets were stratified such that each group had similar ratios of normal to abnormal gaits. All computations were performed on a system with a dual-core 2.7 GHz Intel Core i5-5257U and 8 GB DDR3 memory.

### 2.3. Data Availability

Computer code for all analyses is available at https://gitlab.com/jonyoung/gait_analysis and implemented in Python with the Matplotlib, imbalanced-learn, and scikit-learn libraries [18,19,20].

## 3. Results

The wireless gait analysis sensor (WGAS) collected data from a total of 60 subjects performing six dynamic gait index (DGI) tests. Study demographics are summarized in Table 3. Ten patients were known to have abnormal gaits and the remaining fifty had normal gaits. The patient cohort included adults with or without diagnosed horizontal semicircular canal and otolith dysfunction (vestibular loss). They ranged in age from 21 to 80 years with an average age of 51.8 years. Thirty-eight (63%) of the individuals were female. Subjects walked for a total of 20 feet (6.1 m) while performing gait movements to assess their balance and therefore potential risk for falling, as listed in Table 2 above. As not all subjects completed every possible DGI condition, there were ultimately 319 samples across all subjects performing a particular DGI test, 59 of which corresponded to an abnormal gait.

The range of linear acceleration, $R_{A}$, and range of angular velocity, $R_{\omega }$, in each of the *x*, *y*, and *z* axes (Equations (1) and (2) above) from the output of WGAS worn by each subject represent the six features used to distinguish normal from abnormal gaits. In a clinical setting, the data transmitted by the WGAS is essentially a vector of real-valued voltages, so the minimum and maximum voltages are easily and quickly calculated from the WGAS output. The classifiers trained to predict gait instability are not dependent on the age or other demographic features of the subjects.

### 3.1. Feature Extraction Separates Normal from Abnormal Gaits

**Figure 2:**
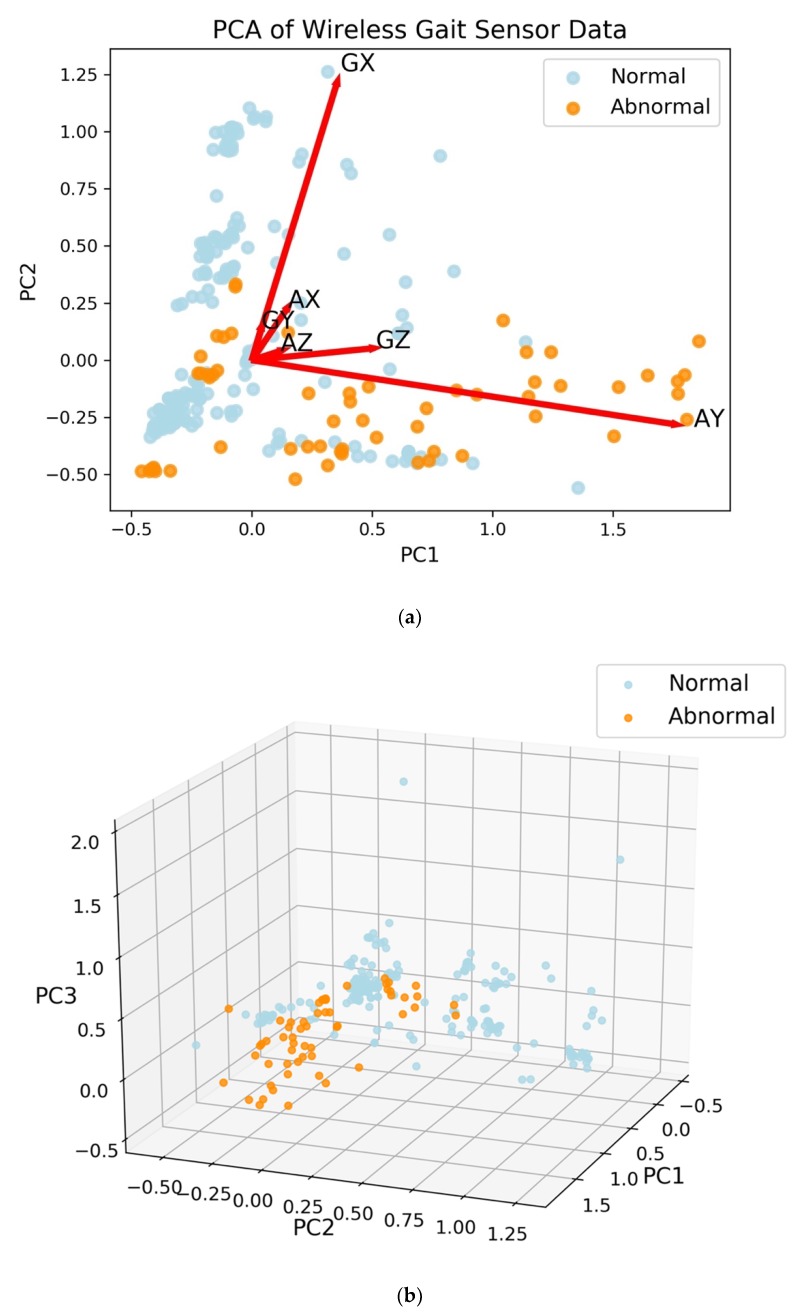
Principal components analysis (PCA) of the wireless gait analysis sensor (WGAS) data. Each point represents a pairing of subject with dynamic gait index test. (**a**) Projection with 2 principal components (PCs) shown with loadings. The first letter of the loading vector labels is for angular velocity from the gyroscope (G) or linear acceleration (A), while the second letter of the label denotes axis (X, Y, or Z). (**b**) With the first three PCs, the subjects with normal gaits are separated from those with abnormal gaits.

The first two principal components (PCs) from PCA were able to describe approximately 83% of the variance of the data. The largest loading for the first principal component is 0.94 for AY, which is the linear acceleration on the *y*-axis (i.e., the acceleration along patient’s forward movement direction during the DGI tests), with the next largest loading equal to 0.26 for GZ, the *z*-axis angular velocity (i.e., the angular velocity along the azimuth axis) [12]. The second principal component has the largest loading of 0.95 for GX, the *x*-axis angular velocity. The third principal component has most of the weight on the *x*-axis acceleration (AX) with a loading of 0.74, while the *y*- and *z*-axis angular velocities (GY and GZ) have loadings of 0.46 and 0.39, respectively. With three PCs, 94% of the variance of the data is explained. The projection onto two-dimensional space is shown with loading vectors in Figure 2a. With three PCs, abnormal gaits are separated from normal gaits (Figure 2b).

### 3.2. An Artificial Neural Network Classifies Abnormal and Normal Gaits with High Accuracy

**Figure 3:**
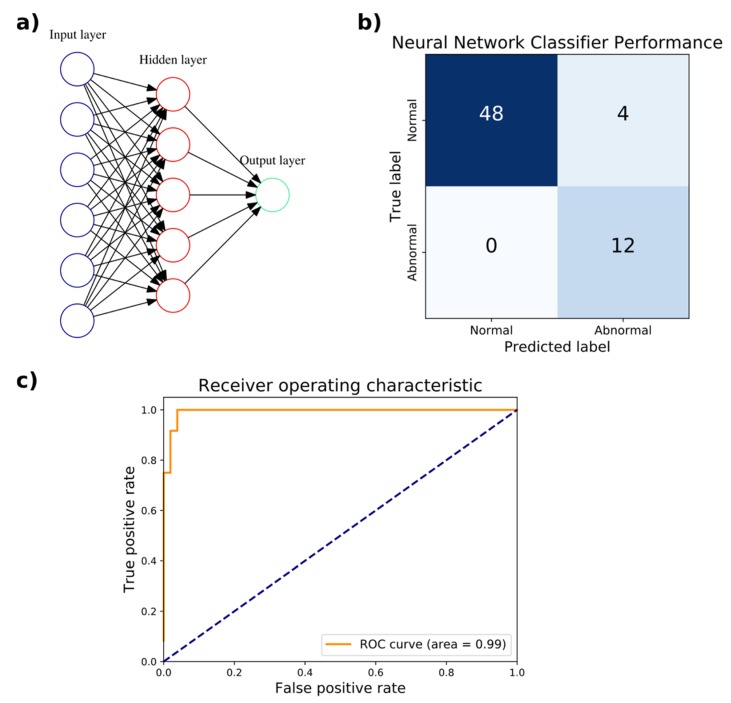
Artificial neural network (ANN) classification of normal versus abnormal gaits measured by the WGAS. (**a**) The optimal ANN architecture for classification with a single hidden layer consisting of five units is depicted. (**b**) The confusion matrix compares the true classification of normal or abnormal gait against the predicted classification. (**c**) The receiver operating characteristic (ROC) curve with the area under the curve (AUC) of 0.99 is shown.

An artificial neural network (ANN) classifier was trained to distinguish normal from abnormal gaits with a random 80–20% train–test split of the WGAS dataset. The training set had 255 samples, 47 of which corresponded to an abnormal gait. The test set contained the remaining 64 samples, with 12 corresponding to an abnormal gait. As described above, each sample corresponds to a subject performing a single DGI test. The training split was further divided randomly 80–20% into a train–validation set for hyperparameter tuning. The input layer has six neurons for the six features from the linear acceleration and angular velocity in the three *x*, *y*, and *z* axes. As a binary classifier, there is only a single output neuron. The hyperparameters optimized through 3-fold cross-validation (CV) were the number of hidden layers and number of neurons in each hidden layer. Based on the accuracy, F_1_ score, and AUC, the optimal hyperparameter chosen was a single hidden layer consisting of five neurons, with an accuracy of 94.7%, F_1_ score of 0.909, and AUC of 1.0. The performance of the hyperparameters evaluated in 3-fold CV is listed in Table 4. On the test set, the accuracy achieved was 93.8% with a sensitivity of 100% and specificity of 92.3%. The AUC of the ROC curve was 0.99 (Figure 3). The neural network classifier was implemented as a scikit-learn MLPClassifier object, which has a memory usage of 56 kilobytes. The wall clock time of the trained model for executing predictions averaged 65 µs over 7 runs of 10,000 iterations each.

### 3.3. A Support Vector Machine Yields Excellent Performance in Gait Classification

**Figure 4:**
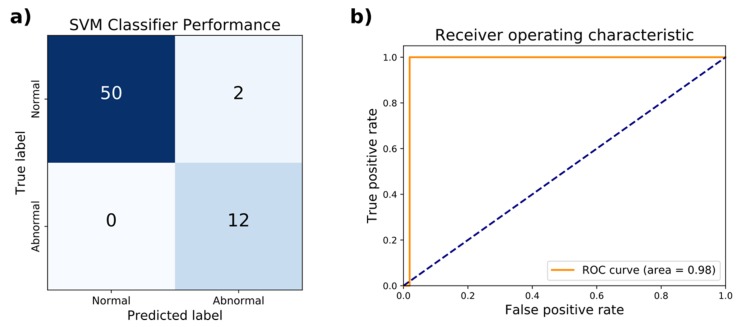
Test set performance of the support vector machine classifier on distinguishing normal from abnormal gaits. (**a**) The confusion matrix comparing the true classification of normal or abnormal gait against the predicted classification is shown. (**b**) The receiver operating characteristic (ROC) curve achieves an area under the curve (AUC) of 0.98.

In addition to ANNs, support vector machines (SVM) were trained to distinguish abnormal versus normal gaits using the identical train and test sets for the ANNs above from the 80–20% split of the WGAS dataset. The penalty parameter of the error term and choice of kernel were among the hyperparameters optimized through 3-fold cross-validation (CV) on the training set. Based on the accuracy and AUC, the optimal hyperparameters chosen were radial basis function with a kernel coefficient γ = 0.1 and penalty parameter of the error term *C* = 1000. In CV, these hyperparameters achieved an accuracy of 96.1% and AUC of 0.968. The performance of the hyperparameters evaluated by 3-fold CV is listed in Table 5. On the held-out test set, the accuracy achieved was 96.9% with a sensitivity of 100% and specificity of 96.1%. The AUC of the ROC curve was 0.98 (Figure 4). The SVM was implemented as a scikit-learn GridSearchCV object, which has a memory usage of 22 kilobytes. The wall clock time of the trained model for executing predictions averaged 129 µs over seven runs of 10,000 iterations each.

### 3.4. Feature Extraction Allows for High-Performance Classification

**Figure 5:**
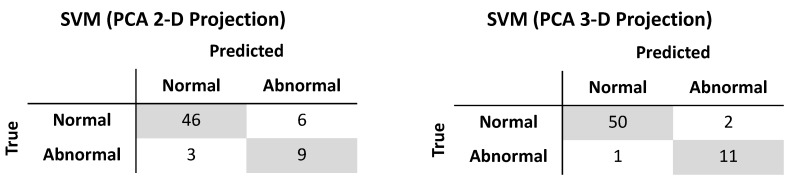
Test set performance of support vector machine classifiers trained after projection by principal components analysis onto two-dimensional (**left**) and three-dimensional (**right**) subspaces.

It was shown earlier that PCA could reduce the dimensionality of the gait features from six to two or three while still capturing 83% or 94% of the variance, respectively. Using similar methods as described above, a radial basis function SVM (γ = 10, C = 10000) trained on the two-dimensional reduced feature set produced an accuracy of 85.9%, sensitivity of 75.0%, and specificity of 88.5% on the test set. When trained on the three-dimensional reduced feature set, the accuracy, sensitivity, and specificity of a radial basis function SVM (γ = 10, C = 100) were 95.3%, 91.7%, and 96.2%, respectively (Figure 5). The wall clock times for test set prediction of the classifiers trained on two and three dimensions averaged 115 µs and 124 µs, respectively, over seven runs of 10,000 iterations each. The memory usage of the classifiers trained on two and three dimensions were 27 and 28 kilobytes, respectively.

## 4. Discussion

In this study, the machine learning techniques achieved successful classification of abnormal and normal gaits in a large cohort of individuals wearing a WGAS. Compared to an ANN classifier, an SVM model demonstrated, in general, somewhat superior performance in terms of overall accuracy, sensitivity, and specificity. All classifiers were evaluated on the same test set. It was vital that a high true negative rate was shown, as clinicians and patients would certainly desire to avoid preemptive treatment for vestibular hypofunction when, in fact, no such pathology was truly present. In a similar vein, a high true positive rate is clearly desirable, as it is important to treat those who definitively have balance problems to mitigate or prevent falls. The patient cohort examined in this study had class imbalance, meaning that there were far more individuals with normal gaits than those with abnormal gaits. Such a distribution may mirror an actual patient population encountered in the clinic. On the other hand, if there had been more subjects with abnormal gaits than those with normal gaits, we would expect ANN and SVM to still yield high classifier performance using the same class balancing techniques from this study. In any case, the SVM and ANN classifiers evaluated here were robust to class imbalance in real-time, after adjusting for such factors. With a larger number of subjects, it is possible that the performance of a newly trained ANN classifier will increase in performance and exceed that of a similarly trained SVM. However, based on the results presented here, an SVM would still be expected to maintain high performance.

Another aspect of predicting individuals at risk involves identifying or engineering the features that are central to the prediction―often referred to as feature selection or feature extraction. Here, PCA on the WGAS data yielded loading vectors that when visualized in two dimensions show the relative contribution or weighting of each feature to the PCs. In the biplot shown in Figure 2, the first PC has most of the weight on the *y*-axis linear acceleration (AY), which is parallel to the direction of travel. The second PC corresponds to GX, which is the angular velocity in the *x*-axis as measured by the gyroscope. Moreover, AY and GX are far apart in the biplot and hence are less correlated with one another. With an even larger clinical sample across more diversified vestibular pathologies and less noise in the data, we expect PCA should be able to provide useful feature extraction from better class separation.

Dimensionality reduction with PCA allowed for classification performance approaching that of the full dimensional data. Specificity of classification upon projection by PCA onto a three-dimensional subspace was identical to classifying on the full dimensional data. The difference in performance came from the sensitivity: when retaining three PCs, only one patient with abnormal gait was misclassified while classification on the full dimensional dataset had perfect sensitivity. As expected, classification performance by SVM increased when retaining three PCs compared to retaining two PCs. Interestingly, dimensionality reduction did not lead to improvements in memory usage, and while execution time of predictions were shorter, the gains were modest: an improvement of 11% and 4% with two and three PCs, respectively. The use of PCA necessitates the storage of the projection matrix to project the test set or any new data onto the lower dimensional subspace, which may account for the observed memory usage.

Previous studies of gait analysis have focused on identifying phases and defining events in walking motions [4,5,6,7,8,9]. For example, Ihlen et al. derived phase-dependent measures of local dynamic stability from 3-D acceleration data on activities of daily living over three days. Using a partial least-squares discriminant analysis, they were able to classify fallers and nonfallers with an AUC ranging from 0.83 to 0.93, depending on the features included in classification [21]. In another approach, Bizovska et al. also employed inertial sensors and demonstrated improved predictive power for fall risk (AUC 0.755) when combining clinical features with measures of local dynamic stability, compared to local dynamic stability alone (AUC 0.673) [22]. Hemmatpour et al. utilized the accelerometer and gyroscope sensors built into smartphones to predict abnormal gait with high accuracy (93.5%) [23]. Here, the feasibility of utilizing direct measurements in real-time to extract simple features (ranges of linear acceleration and angular velocity) from gyroscopes and accelerometers to classify normal and abnormal gaits is demonstrated. Our SVM classifier achieved high predictive performance (96.9% accuracy, 100% sensitivity, and 96.1% specificity). Other studies have also focused on employing such features in gait classification, but have only focused on a small number of patients [13]. In order for fall prediction to transition to clinical use, a larger cohort of patients must be studied. Sixty patients participated under various DGI tests in this study to yield more than 300 gait samples, far greater than the seven patients previously examined, yet the SVM and ANN here still mirrored a similar degree of classification performance [13]. In [13], ANNs outperformed SVMs, while here we find SVMs to be the superior classifier. The SVM has lower memory usage than the ANN while taking longer to perform predictions. Both classifiers have minimal memory requirements and are capable of performing real-time gait classification from WGAS. Yet the issue of feature selection of extraction in gait measurement still remains. Howcroft et al. evaluated various feature selection techniques, including correlation-based feature selection, fast correlation-based filter, and Relief-F, and utilizing a SVM classifier achieved 78% accuracy, 26% sensitivity, and 95% specificity [24]. Feature extraction through PCA is shown here to potentially resolve the features that contribute most to differentiate normal from abnormal gaits, as using PCA as a preprocessing step for SVM yielded high predictive performance (95.3% accuracy, 91.7% sensitivity, and 96.2% specificity). More studies are needed to address vestibular hypofunction in the geriatric population and characterize the benefit of a gait analysis sensor in detecting gait differences in this population [25,26]. As a step in this direction, we have included older age individuals in our study. We also quantified vestibular hypofunction using clinical objective testing that allows one to diagnose even mild vestibular dysfunction that may result in functional gait disruptions.

While this study reports the prediction of abnormal from normal gaits on a large patient cohort, clinical trials will be necessary to further transition machine learning-based fall risk prediction into the clinic, particularly as asymmetric peripheral vestibular function does not necessarily imply balance deficit or pathology. The results of this study are preliminary as the methodology described here will need to be further validated on an even larger number of patients. Furthermore, predicting and testing patients for vestibular hypofunction does not necessarily cover all mechanisms of falling, for example from muscular, neurologic or visual deficits. In the future, it may be interesting to perform a train–test split for machine learning such that an individual’s data for all six DGI tests are kept entirely together in either the train or test set, instead of possibly being split across both. Such an approach would be relevant to the case when patients are evaluated in the clinic by conducting all DGI tests together. It would also be interesting to move beyond binary predictions (normal vs. abnormal) to continuous predictive scores and investigate fall risk stratification according to the predicted probability of falling. Moreover, the performance of other statistical learning models other than SVMs or ANNs would be informative, such as with dynamic time warping on WGAS data, given its applicability to time series and use in varied fields from finance to medicine [27]. Future directions may include comparison of WGAS fall risk assessment with standard clinical vestibular testing. With an aging demographic in the United States, the potential of reducing and preventing fall injuries provides a compelling application for brining data-driven predictive analytics into the clinic.

## Figures and Tables

**Table 1:** Specifications of the wireless gait analysis sensor.

Approximate Range of Operation	12 m (40 ft)
Approximate battery life	40 h (estimated for always-on time)
Weight	42 g
Dimensions	2.2” × 1.5” × 0.8”

**Table 2:** Conditions of dynamic gait index (DGI) movements.

Condition	Description
1	Walk on a level surface at normal speed for 20′
2	Walk at normal speed for 5′, walk fast for next 5′, walk slowly for next 5′, walk at normal speed for final 5′
3	Walk for 20′ while turning the head horizontally
4	Walk for 20′ while turning the head vertically
5	Walk normally up to the 20′ mark; at the end pivot to turn around
6	Walk normally for 20′ with an obstacle in the path; step over (not around) the obstacle

**Table 3:** Summary of study demographics.

	Male	Female	Normal Gait	Abnormal Gait
**Number of subjects**	22	38	50	10
**Range**	21–80 years old			
**Average age**	51.8 years old			

**Table 4:** Hyperparameter tuning of artificial neural network training.

Number of Hidden Layers	Number of Neurons Per Hidden Layer	Accuracy	F_1_ Score	AUC
1	3	0.895	0.800	0.986
1	4	0.895	0.800	0.986
**1**	**5**	**0.947**	**0.909**	**1.000**
1	6	0.895	0.800	0.986
1	7	0.947	0.909	0.986
1	8	0.947	0.909	0.986
2	3/2	0.263	0.417	0.757
2	4/2	0.263	0.417	0.857
2	4/3	0.895	0.800	0.986
2	5/2	0.947	0.909	0.943
2	5/3	0.263	0.417	0.786
2	5/4	0.263	0.417	0.757

**Table 5:** Hyperparameter tuning of support vector machine classifier training.

Kernel	Hyperparameters	Accuracy	AUC
Linear	*C* = 10	0.910	0.936
Linear	*C* = 10^2^	0.933	0.944
Linear	*C* = 10^3^	0.937	0.941
Linear	*C* = 10^4^	0.941	0.941
Linear	*C* = 10^5^	0.937	0.938
**Radial basis function**	***C* = 10^3^, γ = 10^−1^**	**0.961**	**0.968**
Radial basis function	*C* = 10^3^, γ = 10^−2^	0.918	0.944
Radial basis function	*C* = 10^3^, γ = 10^−3^	0.878	0.922
Radial basis function	*C* = 10^4^, γ = 10^−1^	0.961	0.964
Radial basis function	*C* = 10^4^, γ = 10^−2^	0.914	0.942
Radial basis function	*C* = 10^4^, γ = 10^−3^	0.922	0.945
